# Helminth Excreted/Secreted Antigens Repress Expression of LPS-Induced Let-7i but Not miR-146a and miR-155 in Human Dendritic Cells

**DOI:** 10.1155/2013/972506

**Published:** 2012-12-27

**Authors:** Luis I. Terrazas, Fausto Sánchez-Muñoz, Magaly Pérez-Miranda, Ana M. Mejía-Domínguez, Yadira Ledesma-Soto, Rafael Bojalil, Lorena Gómez-García

**Affiliations:** ^1^Biomedicine Unit, Facultad de Estudios Superiores Iztacala, UNAM, Avenida de los Barrios No. 1 Col. Los Reyes Iztacala, 54090 Tlalnepantla, MEX, Mexico; ^2^Department of Immunology, Instituto Nacional de Cardiología Ignacio Chávez, Juan Badiano No. 1 Col. Sección XVI, 140080 Tlalpan, DF, Mexico; ^3^Blood Bank, Instituto Nacional de Cardiología Ignacio Chávez, Juan Badiano No. 1 Col. Sección XVI, 140080 Tlalpan, DF, Mexico; ^4^Department of Health Care, Universidad Autónoma Metropolitana Xochimilco, Calzada del Hueso No. 1100 Col. Villa Quietud, 04960 Coyoacán, DF, Mexico

## Abstract

MicroRNAs have emerged as key regulators of immune responses. They influence immune cells' function and probably the outcome of several infections. Currently, it is largely unknown if helminth parasites and their antigens modify host microRNAs expression. The aim of this study was to explore if excreted/secreted antigens of *Taenia crassiceps* regulate LPS-induced miRNAs expression in human Dendritic Cells. We found that these antigens repressed LPS-let-7i induction but not mir-146a or mir-155 and this correlates with a diminished inflammatory response. This let-7i downregulation in Dendritic Cells constitutes a novel feature of the modulatory activity that helminth-derived antigens exert on their host.

## 1. Introduction

MicroRNAs (miRNAs) are small (~23 nucleotides) noncoding RNAs that negatively regulate protein-coding gene expression mainly via down-regulation of mRNA levels and/or translational suppression [1]. In the last years, several reports have linked miRNAs to multiple and essential functions in the immune system. These molecules have emerged as important regulators of the development and differentiation of T and B lymphocytes and dendritic cells (DCs) [2–4], as modulators of inflammation [5], of balance between Th1 and Th2 responses [6], and of antibody production [7] amongst other functions. Besides their physiological role, miRNAs participate in pathological aspects of the immune response; for example, these molecules may be important mediators in cancer and autoimmunity [8] but participate as well in the control of viral infections [9]. The response of host miRNAs in parasitic infections is largely unknown. The few reports that have addressed the influence of parasites upon host-derived miRNAs have focused on protozoan infections [10, 11] while the role of helminth parasites and their antigens as possible modulators of such miRNAs is widely unexplored. 

Dendritic Cells (DCs) dictate immune responses through the different signals derived from them such as secreted cytokines, chemokines, and also costimulatory molecules. Once an immature DC faces stimuli, either exogenous or endogenous, this cell will undergo an intracellular process that ultimately will render it capability of supporting the different types of immune responses [13]. DCs exposed to lipopolysaccharide (LPS) are cells that suffer a typical maturation process with secretion of inflammatory cytokines and chemokines mainly IL-12, TNF, IL-6, RANTES, MCP-1 among others, and an upregulation of certain membrane molecules. Altogether, these changes confer DCs with the capability to induce the appropriate adaptive immune responses [14, 15]. Recently, it has been described that some miRNAs are upregulated and may participate in these dendritic cell's maturation/activation events [16, 17]. Specifically, miR-146a, miR-155, and let-7i are microRNAs that are modulated positively when DCs are exposed to maturation agents including LPS, TNF, and IFN-*γ* [18, 19] and seem to be related to the activation events triggered in DCs by such stimuli including the expression of costimulatory molecules, secretion of proinflammatory cytokines, and even induction of apoptosis [16–18]. Although there is one report showing that chronic ascariasis and trichuriasis modify the expression of miRNA let-7d in peripheral-blood mononuclear cells (PBMCs) [20], the impact of helminthes and their antigens on DCs-derived miRNAs and its possible consequences for the function of these cells has not been addressed to date. 

We recently demonstrated that some properties of human DCs can be affected by their exposure to the excreted/secreted antigens derived from the cysticerci of *Taenia crassiceps *(TcES) [12]. These cells were characterized by an immature phenotype with low expression of the molecules CD80, CD86, and CD83 and were capable of secreting the regulatory cytokine IL-10 but not any of the inflammatory cytokines tested. More importantly, DCs exposed to TcES showed impaired maturation/activation to subsequent LPS stimulation, where both expression of costimulation molecules and secretion of proinflammatory cytokines were significantly diminished, indicating the anti-inflammatory effects of TcES upon this cell type [12]. Since TcES affects events related with the activation of DCs by LPS, here we explored if these parasite antigens modify the expression in human DCs of the microRNAs miR-146a, miR-155, and let7i, which, as stated earlier, seem to play a critical role in the classical maturation and activation processes induced by LPS in these cells.

## 2. Methods

### 2.1. *Taenia crassiceps* Excreted/Secreted Antigens

Metacestodes of *Taenia crassiceps* were harvested from the peritoneal cavity of female Balb/c mice after 2–4 months of infection. This was done under a laminar flow chamber and sterile 1X PBS was used. The cysticerci were washed four times with 1X PBS and maintained in culture in 1X PBS at 37°C for 24 h. TcES were recovered from the supernatant and centrifuged for 10 min at 1000 g using LPS-free filters. This fraction was concentrated using 50 kDa Amicon Ultra Filter (Millipore). Concentrations of different lots range between 400 and 920 ug/mL. Samples were stored at −70°C until further use.

### 2.2. Monocyte-Derived Dendritic Cells

Human peripheral blood mononuclear cells (PBMCs) were obtained from buffy coats of 12 healthy blood donors from the Instituto Nacional de Cardiología Ignacio Chávez's Blood Bank. Informed consent was obtained for the use of blood samples according to the declaration of Helsinki and the local scientific and ethics committees approved the protocol. PBMCs were isolated by Ficoll-gradient centrifugation (GE Healthcare), analyzed in a Coulter AcT for cellular types (Beckman Coulter), and 3 × 10^6^ monocytes were left to adhere in 6-well culture plates for 2 h. After this period, nonadherent cells were washed away and adherent cells were cultured in RPMI medium supplemented with 10% SFB and penicillin/streptomycin in presence of 400 U/mL of IL-4 and 800 U/mL of GM-CSF during 6 days with replacement of medium and cytokines at day 3. At day 6 nonadherent cells were recovered and placed for 24 h in fresh medium. At this point we determined by flow cytometry the percentage of CD11c+ cells and for all experiments this was ≥80%. Cells were challenged with 20 ug/mL TcES, 1 ug/mL LPS, or a combination of them for 3 or 24 h. Control cells received RPMI. 

### 2.3. QRT-PCR for Mature MicroRNAs

To asses mature microRNA expression, we used two-step qRT-PCR, with TaqMan microRNA assays (Applied Biosystems). For microRNA cDNA synthesis, a 15 *μ*L reaction volume, composed of 1.5 *μ*L of Buffer (10x), 0.15 *μ*L 100 mM dNTPs (100 mM), 1.0 *μ*L reverse transcriptase, 0.19 *μ*L RNAse inhibitor (20 U/*μ*L), and 3.0 *μ*L of each specific microRNA primer, was mixed with 10 ng of total RNA. RT reaction was incubated 30 minutes of 16°C, 30 minutes of 42°C, and 5 minutes of 85°C. Duplicate real-time PCR reactions were performed in a Roche LigthCycler 2.0. Reaction mix was composed of 1x LigthCycler TaqMan Master (Roche), 1X of each specific microRNA probe, and 2.5 uL of specific microRNA cDNA (diluted 1 : 3). These were followed by 10 minutes 95°C for preincubation, 40 cycles of amplification program consisting of 95°C 10 sec, 60°C 40 sec, and 72°C 5 sec (fluorescence acquisition). To asses possible bias for reference gene selection U6 and let7a were used as reference genes, and relative quantification was calculated by the formula 2-(Cp microRNA target—Cp U6 or let7a). All microRNA assays were tested for reproducibility and linearity (PCR efficiency was between 1.9 and 2.0 for all assays).

### 2.4. Quantification of Cytokine Production

Supernatants were recovered after 3 h and 24 h stimulation period and production of the cytokines TNF, IL-6, IL-12, IL-10 (Peprotech), and MCP-1 (R&D) was measured by ELISA kits in the supernatants of DCs cultures. For the analysis of cytokine mRNAs levels cells were recovered from cultures 3 h after stimulation.

### 2.5. Statistics

Comparisons were performed by the Mann-Whitney Wilcoxon test for cytokine comparisons and Kruskal-Wallis and Dunn's *post hoc* test for miRNAs expression. Significance was set on a *P* value <0.05. All analyses were performed with the GraphPad Prism v. 5 statistical software. 

## 3. Results and Discussion 

### 3.1. TcES Dampen the Inflammatory Activity of LPS in Human DCs

LPS activation of DCs induces their maturation and secretion of pro-inflammatory cytokines and chemokines such as IL-12, TNF, IL-6, MCP-1 among others [11, 13]. We demonstrated previously that TcES does not induce secretion of the pro-inflammatory cytokines IL-12, IL-1*β*, TNF, or IL-6 but contrary it upregulates the secretion of IL-10. More importantly, these cysticerci-derived antigens interfered with the LPS-activation of DCs, downmodulating their maturation, and reversing the secretion of all the pro-inflammatory cytokines tested but not the one of regulatory cytokine IL-10 [12].

**Figure 1:**
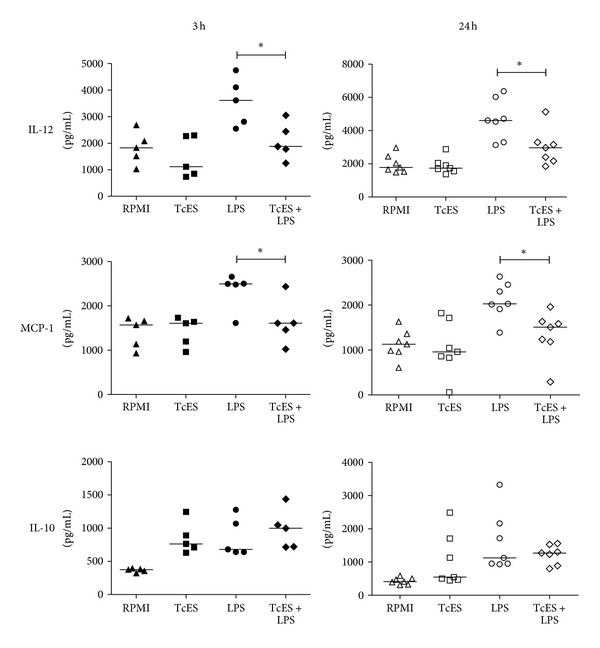
TcES modify the secretion of cytokines by human DCs activated with LPS. Human peripheral blood mononuclear cells (PBMCs) were obtained from buffy coats of 12 healthy blood donors. Informed consent was obtained for the use of blood samples according to the declaration of Helsinki and the local scientific and ethics committees approved the protocol. Monocyte-derived DCs were cultured for six days in the presence of IL-4 and GM-CSF [12]. Cells were challenged with 20 ug/mL TcES, 1 ug/mL LPS, or a combination of them for 3 h or 24 h. Control cells received RPMI. Cells and supernatants were collected and cytokine response was measured by ELISA kits (Peprotech and R&D). The data is shown as scattered plots and medians of twelve independent experiments (*n* = 5 for 3 h and *n* = 7 for 24 h). Comparisons were performed by the Mann-Whitney test. *P < 0.05 LPS *versus* LPS/TcES. All analyses were performed with the GraphPad Prism v. 5 statistical software.

We tested if the phenomenon of modulation of TcES upon LPS-induced cytokine response was observed in the monocyte-derived DCs of the donors used for this study. At day six of culture, DCs received TcES, LPS, or a combination of both for 3 h and 24 h, and supernatants were recovered. As expected, we found that unlike LPS, TcES did not induce secretion of the inflammatory molecules IL-12 and MCP-1 (Figure 1) but instead, these antigens were capable of downmodulating such response to LPS both at 3 h or 24 h after stimulation (Figure 1). Interestingly, secretion of the regulatory cytokine IL-10 was induced similarly by TcES, LPS, and the combination of both (Figure 1). Altogether, these results indicate that these helminth antigens promote human DCs to acquire an anti-inflammatory phenotype while dampening the inflammatory response induced by LPS. These observations allowed us to establish that the cells tested for miRNAs expression were indeed consistently modulated by TcES as reported previously by our group [12].

### 3.2. TcES Do Not Modify Expression of Mir-146 and Mir-155 but Downregulate Expression of Let-7i

miRNAs control expression of genes through mRNA degradation or translational suppression [1]. The miRNAs mir-146a, mir-155, and let-7i have been reported to play a role in the induction of maturation and activation of DCs [14–17]. Currently, it is unknown if helminth parasites or their antigens exert a modulatory activity on these host miRNAs. Since in a previous work we found that TcES affects the maturation and secretion of pro-inflammatory cytokines in DCs activated with LPS [12], we explored if these antigens affect the expression of the LPS-induced mir-146a, mir-155, and let-7i in human DCs. 

**Figure 2:**
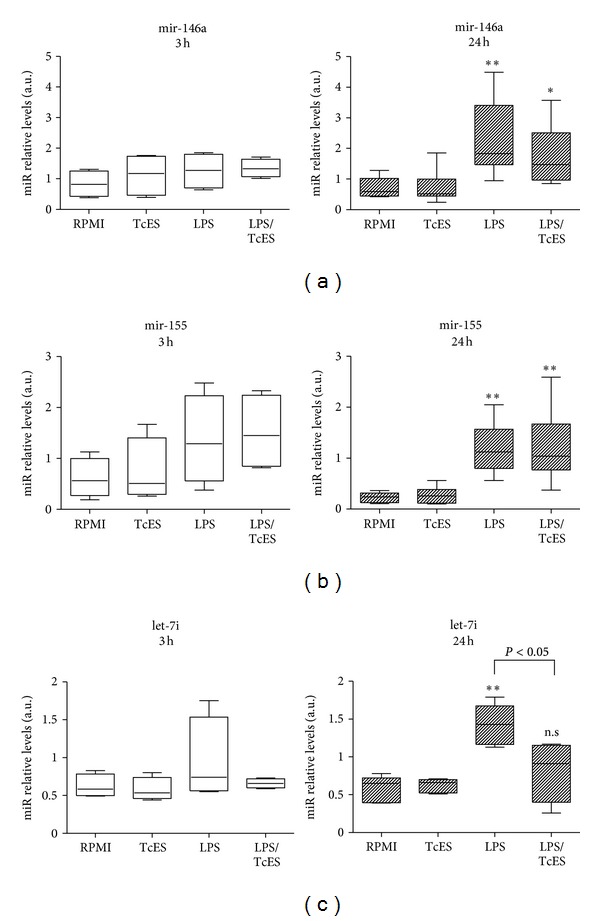
Expression of miRNAs mir-146a, mir-155, and let-7i in human DCs activated with LPS and exposed to excreted/secreted *T. crassiceps* antigens. The miRNAs expression was evaluated after stimulation of human DCs with 20 ug/mL TcES, 1 ug/mL LPS, or a combination of them for 3 or 24 h. Control cells received RPMI. Determination of miRNAs relative levels by qRT-PCR was conducted using mature miRNAs specific TaqMan assays in cultures from nine independent experiments (4 donors for 3 h and 5 donors for 24 h). The gene *let-7a* was used as reference gene and relative quantification was calculated by the formula 2^−(CT  targetmiRNAs−CT  reference)^. All miRNAs assays were tested for reproducibility and linearity (PCR efficiency was between 1.9 and 2.0 for all assays). The data is shown as boxplot, horizontal line denotes the median value, box encompasses the upper and lower quartiles and the whiskers, and the minimum and maximum data value. The relative expression values were analyzed using the nonparametric Kruskal-Wallis test and Dunn's *post hoc *comparisons. For (a) y (b) *P < 0.05 and **P < 0.01  *versus *RPMI control group were deemed significant. For (c) *P < 0.05 for LPS *versus* LPS/TcES and n.s. for LPS/TcES *versus* RPMI control group. All analyses were performed with the GraphPad Prism v. 5 statistical software.

qRT-PCR was performed in independent DCs culture experiments of nine healthy donors (*n* = 4 for 3 h and *n* = 5 for 24 h) and two reference genes (*u6* and *let-7a*) were used to establish fold-induction of miRNAs. As already described by others [18, 19], we found that at 24 h after stimulation of DCs, LPS upregulated the expression of mature mir-146a, mir-155, and let7i when compared to cells receiving medium alone (Figure 2). Exposure of DCs to TcES alone did not induce an upregulation on the three selected miRNAs (Figure 2) but when these cells were exposed to the combination of TcES plus LPS we observed a contrasting response. Prior to such combined stimuli, we found no significant differences in the expression of mir-146a and mir-155 (Figures 2(a) and 2(b)), indicating that TcES do not modulate the expression of these two miRNAs in human DCs activated by LPS. However, when we assessed the influence of such antigens over the LPS-induced expression of let-7i, the upregulation of this miRNA was no longer observed: statistical difference was found between LPS and LPS/TcES whereas none was found between LPS/TcES and RPMI (Figure 2(c)). At 3 h after stimulation, the expression of none of the selected miRNAs was statistically significant between groups (Figure 2). Results with the reference gene *U6* were found similar to those with let-7a (data not shown). Altogether, these data show that the helminth-derived antigens TcES importantly interfere with the LPS-induced expression of let-7i in human DCs and suggest that such antigens prevent the upregulation of this miRNA rather that diminishing it after a prior augmentation. TcES appear to exert a fine modulatory activity upon host miRNAs since even when mir-146a and mir-155 have also been related to the LPS-induced response in DCs, only let-7i was affected by such antigens. Thus, this is the first evidence showing that antigens derived from a helminth parasite, indeed, possess modulatory activities over the expression of miRNAs in human DCs.

The lethal-7 (let-7) family of miRNAs has been linked to important aspects of the immune response, such as inflammation, autoimmunity, and host-pathogen interactions [21, 22]. In particular, the expression of its member let-7i seems to be affected by some protozoans like *Cryptosporidium parvum* and *Plasmodium berghei *and in turn possibly affecting the immune response against them [23, 24]. More importantly, let-7i is up-regulated in DCs in response to LPS and participates in the induction of their maturation and secretion of proinflammatory cytokines such as IL-12 [17, 18]. Interestingly, secretion of IL-10 is only favored when let-7i is inhibited [17], indicating the inflammatory activity that this miRNA possesses on DCs activated by LPS. Here we show that TcES downmodulate the LPS-induced expression of let-7i in human DCs in parallel to a diminished inflammatory cytokine/chemokine profile and a normal secretion of IL-10. Thus, it is likely that the previously reported impaired maturation and the repeatedly observed anti-inflammatory phenotype of DCs exposed to TcES can be due to the repressing effects that these antigens possess upon let-7i. Moreover, it has been shown that one of the targets of let-7i is the suppressor of cytokine signaling 1 (SOCS1) [17], a molecule that has been related to the downregulation of DC maturation and secretion of the pro-inflammatory cytokines IL-12, IL-6, and IFN-*γ* [25, 26]. Even when we did not assess the expression of SOCS1 in human DCs exposed to TcES, we hypothesize that the downregulation of let-7i by these antigens may in turn affect the levels of SOCS1 regulating in this manner the maturation and the inflammatory cytokine profile of such DCs. The mechanisms involved in the modulation of TcES on let-7i expression in human DCs are currently unknown. Interestingly, O'Hara and colleagues showed that *C. parvum* parasite decreases let-7i expression by promoting the formation of a NF-*κ*B p50-C/EBP*β* silencer complex in cholangiocytes [27]. If this is the mechanism involved in the repression of let-7i expression by the helminth antigens used in our study remains to be determined.

In conclusion, TcES are helminth-derived antigens that exert modulatory effects upon the expression of the LPS-induced miRNA let-7i. It is possible that this downmodulation underlies the previously reported dampening of maturation in human DCs activated with LPS and the diminished secretion of pro-inflammatory cytokines observed again in this study. To our knowledge, this is the first report that assesses the possible regulatory actions of helminth antigens upon host miRNAs. The downregulation of let-7i by TcES opens a new route in the study of modulation of the host response by *T. crassiceps *and possibly other helminths. This study establishes the basis for the implement of functional studies where silencing and overexpression of miRNAs may allow us to elucidate the complex mechanisms involved in the regulatory activities that helminth antigens possess over the immune system.
